# Species-specific control of acoustic gaze by echolocating bats, *Rhinolophus ferrumequinum nippon* and *Pipistrellus abramus*, during flight

**DOI:** 10.1007/s00359-016-1121-0

**Published:** 2016-08-26

**Authors:** Yasufumi Yamada, Shizuko Hiryu, Yoshiaki Watanabe

**Affiliations:** 1 Faculty of Life and Medical Sciences, Doshisha University, Kyotanabe, 610-0321 Japan; 2 JST, PRESTO, 4-1-8 Honcho, Kawaguchi, Saitama 332-0012 Japan

**Keywords:** Pulse direction, Beam width, Acoustical field of view, Microphone array, Telemetry microphone

## Abstract

Based on the characteristics of the ultrasounds they produce, echolocating bats can be categorized into two main types: broadband FM (frequency modulated) and narrowband CF (constant frequency) echolocators. In this study, we recorded the echolocation behavior of a broadband FM (*Pipistrellus abramus*) and a narrowband CF echolocator species (*Rhinolophus ferrumequinum nippon*) while they explored an unfamiliar space in a laboratory chamber. During flight, *P. abramus* smoothly shifted its acoustic gaze in relation to its flight direction, whereas *R. ferrumequinum nippon* frequently shifted its acoustic gaze from side to side. The distribution of the acoustic gazes of *R. ferrumequinum nippon* was twice as wide as that of *P. abramus*. Furthermore, *R. ferrumequinum nippon* produced double pulses twice as often as *P. abramus*. Because *R. ferrumequinum nippon* has a horizontal beam width (−6 dB off-axis angle) half as wide (±20.8 ± 6.0°) as that of *P. abramus* (±38.3 ± 6.0°), it appears to double the width of its acoustical field of view by shifting its acoustic gaze further off-axis and emitting direction-shifted double pulses. These results suggest that broadband FM and narrowband CF bats actively control their acoustic gazes in a species-specific manner based on the acoustic features of their echolocation signals.

## Introduction

Echolocating bats are acoustically guided animals that emit ultrasound pulses and perceive space by analyzing the returning echoes, a form of active sensing without visual input. Echolocation can be categorized into two main types based on the spectral and temporal characteristics of emitted sounds. Rhinolophidae, Hipposideridae, and some species of Mormoopidae are narrowband constant frequency (CF) echolocators, emitting long calls dominated by a single CF separated by brief periods of silence (i.e., high duty cycle, HDC) (Fenton et al. 2012). Narrowband CF bats employ Doppler-shift compensation (DSC), adjusting the frequency of their calls to maintain echo frequency within the acoustic fovea, thus avoiding masking effects using frequency to separate pulses and their echoes (Schnitzler 1968; Schnitzler and Denzinger 2011). Broadband frequency-modulated (FM) bats, on the other hand, emit broadband pulses of short duration, with long intervals of silence between them, using time rather than frequency to separate pulses and echoes and avoid masking effects (i.e., low duty cycle, LDC). Narrowband CF and broadband FM echolocation differ in a number of respects, and bats from each group are thought to adapt their acoustic and flight behaviors according to their foraging habitats (Simmons and Stein 1980; Neuweiler 1984; Fenton 2010, 2013).

Using a directional beam of ultrasound to echolocate in the air, where sounds are quickly attenuated, bats rely on beamforming to optimize their acoustical field of view. The beam directionality of echolocation pulses has been evaluated in several types of bats, such as the narrowband CF species *Rhinolophus ferrumequinum* (Grinnell and Schnitzler 1977), *Hipposideros terasensis* (Hiryu et al. 2006), and *Pteronotus parnellii* (Hartley and Suthers 1990) and the broadband FM species *Eptesicus fuscus* (Hartley and Suthers 1989), *Carollia perspicillata* (Hartley and Suthers 1987), and *Myotis* (Shimozawa et al. 1974). Recently, microphone arrays have been used to measure echolocation beam directionality during flight in both field and laboratory settings (i.e., Surlykke et al. 2009b; Jakobsen and Surlykke 2010; Jakobsen et al. 2013, 2015). These studies have reported that bats actively adjust their beam width according to the situation. Mouth-emitting FM bats have been observed to adjust their mouth gape to optimize their acoustical fields of view, using a narrow beam width when entering a confined space and widening it as they approach more open spaces (Surlykke et al. 2009b; Kounitsky et al. 2015). Active beam adjustment is also found in the narrowband CF bat *R. ferrumequinum nippon,* a nostril-emitting species, during prey-capture flight in a laboratory setting (Matsuta et al. 2013). During the final stages of prey capture, *R. ferrumequinum nippon* actively expands its beam width without changing its call frequency to retain a moving target within its acoustic field of view. Such dynamic beam control is considered a common behavioral strategy among both broadband FM and narrowband CF bats.

Most broadband FM bats emit echolocation pulses through their mouths. The beam directionality of mouth-emitting species can be modeled as a circular piston in an infinite baffle, with the parameter of diameter determined by the size of the mouth opening (Strother and Mogus 1970; Mogensen and Møhl 1979; Jakobsen and Surlykke 2010; Jakobsen et al. 2013). Based on the acoustical principle of the circular piston model, directivity is determined by the interaction of mouth aperture and wavelength (i.e., if aperture size remains constant, higher call frequencies create a narrower beam). Jakobsen et al. (2013) found that six aerial hawking vespertilionid species with different body sizes produced calls at different frequencies, with smaller bats emitting high-frequency calls. The different frequencies created sonar beams with extraordinarily similar patterns of directivity, following the rule that if frequency remains constant, decreasing emitter size creates a narrower the beam. This finding indicates that the bats adjust their calls to create similar acoustic fields of view under similar conditions, regardless of body size.

Acoustic scanning is another important behavioral mechanism used by echolocators to determine their acoustical field of view and explore their environment. Acoustic gaze, defined as the angular difference between the directions of flight and pulse emission, has been experimentally investigated in flying bats in both field (Fujioka et al. 2014; Seibert et al. 2013, 2015) and laboratory settings (Ghose and Moss 2003; Ghose et al. 2006; Surlykke et al. 2009a; Kinoshita et al. 2014). Using acoustic scanning, flying *Eptesicus fuscus* can aim their directional beam to within 3° of a stationary target (Ghose and Moss 2003). We have previously shown that *Rhinolophus ferrumequinum nippon* approaching a moving moth in flight in a laboratory chamber can track its target to within less than 5° (Matsuta et al. 2013). Acoustic gaze is a useful index of bats’ attention when selecting targets, and it is analogous to gaze control in visually guided animals. Interestingly, *Eptesicus fuscus* has been observed to conduct sequential gaze shifts between multiple objects when simultaneously performing tasks involving obstacle avoidance and prey capture (Surlykke et al. 2009a). Such sequential gaze shifting has also been reported in the free-swimming harbor porpoise *Phocoena phocoena* when discriminating between two targets (Wisniewska et al. 2012) and in flying *R. ferrumequinum nippon* when choosing between two moths (Kinoshita et al. 2014). These studies demonstrate that echolocating animals control the flow of spatial and temporal information by adjusting not only beam width but also gaze control.

Furthermore, *Eptesicus fuscus* produces sonar sound groups (i.e., double pulses) more often in complex environments or when performing complicated tasks ( Moss et al. 2006; Kothari et al. 2014; Warnecke et al. 2016). The emission of sonar sound groups has been reported in a number of bat species, which suggests that exercising temporal control over emissions helps bats negotiate complex or unfamiliar environments (Moss and Surlykke 2010). In particular, it was suggested that the emission of double pulses allows bats to have immediate and more detailed surrounding information for planning flight paths (Moss et al. 2006) or for improving the resolution of an uncertain target’s position (Kothari et al. 2014).

As just described, bat species vary in their vocalization characteristics, such as frequency band, interpulse interval, and intensity, which may result in species-specific gaze control strategies and practical beam directionalities. Because narrowband CF species are bigger and emit calls with higher dominant frequencies than do broadband FM species, they use narrower sonar beams, which likely affect scanning behavior during echolocation. Therefore, we hypothesized that CF and FM bats will display species-specific behavioral strategies to control acoustical gaze based on their particular beam directionality to adjust the sensory volume of echo information when navigating complex environments. In this study, we compared the echolocation behaviors of two bat species, *Pipistrellus abramus* (a broadband FM species) and *Rhinolophus ferrumequinum nippon* (an narrowband CF species), by measuring pulse direction and beam width during free flight in a flight chamber. We used naïve subjects, focusing on behavior during their first flight in the chamber so that we could investigate the optimum performance of beam-sight and pulse-direction controls during spatial scanning in an unfamiliar space. In brief, we investigated whether the use of pulse direction is affected by the species-specific beam widths of echolocation pulses.

## Materials and methods

### Subjects

Seven adult Japanese house bats (*Pipistrellus abramus*, body length: 4.0–6.0 cm, body mass: 5–10 g) and six adult Japanese horseshoe bats (*Rhinolophus ferrumequinum nippon*, body length: 6.0–8.0 cm, body mass: 20–30 g) were used in this study. *P. abramus* were captured from a large colony roosting in the girders of a bridge near Doshisha University. The animals were kept in a rearing cage (30 × 30 × 20 cm) in a temperature-controlled room and were allowed free access to mealworms and water. *R. ferrumequinum nippon* were captured from natural caves in Hyogo and Osaka Prefectures in Japan. The animals were housed in a temperature- and humidity-controlled colony room [4 m (L) × 3 m (W) × 2 m (H)] at Doshisha University in Kyoto, Japan. The bats were allowed to fly freely and given access to mealworms and water. The day/night cycle of the room was set to 12 h of dark followed by 12 h of light.

**Fig. 1:**
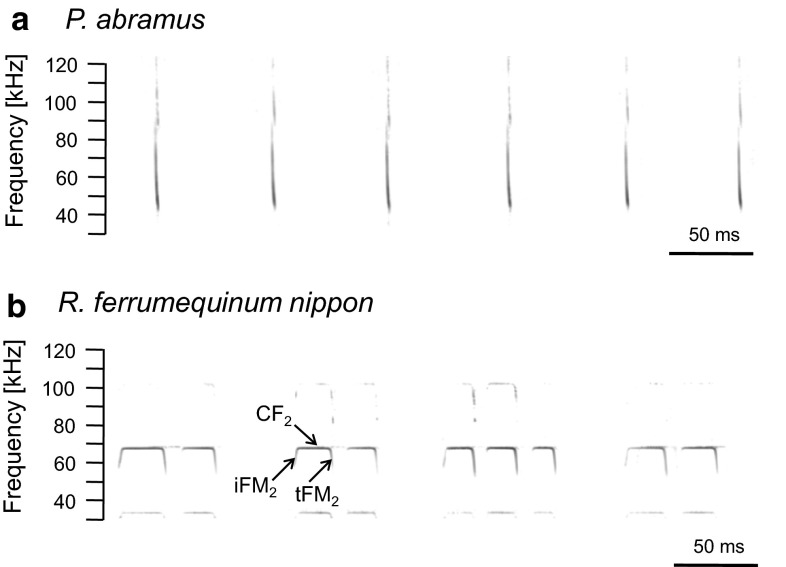
Sonograms of typical pulse emission sequences in *P. abramus* (**a**) and *R. ferrumequinum nippon* (**b**) during flight in the flight chamber. Sounds were recorded by the on-board microphone (Telemike) mounted on the back of each bat

*P. abramus* emit downward FM pulses with harmonics, and the frequency of the fundamental component is exponentially modulated. The means of the initial and terminal frequencies of the fundamental FM sweep were 80–90 kHz and 40–45 kHz, respectively (Fig. 1a). The echolocation pulses emitted by *R. ferrumequinum nippon* are compound signals, each consisting of a CF component plus an accompanying initial short upward FM sweep (iFM_2_: 2–8 kHz, ending at 68–70 kHz) and a terminal short downward FM sweep (tFM_2_: beginning at 68–70 kHz and dropping 8–12 kHz) (Fig. 1b). The second harmonic of the CF component (CF_2_), around 68–70 kHz, is the strongest.

### Experimental conditions

**Fig. 2:**
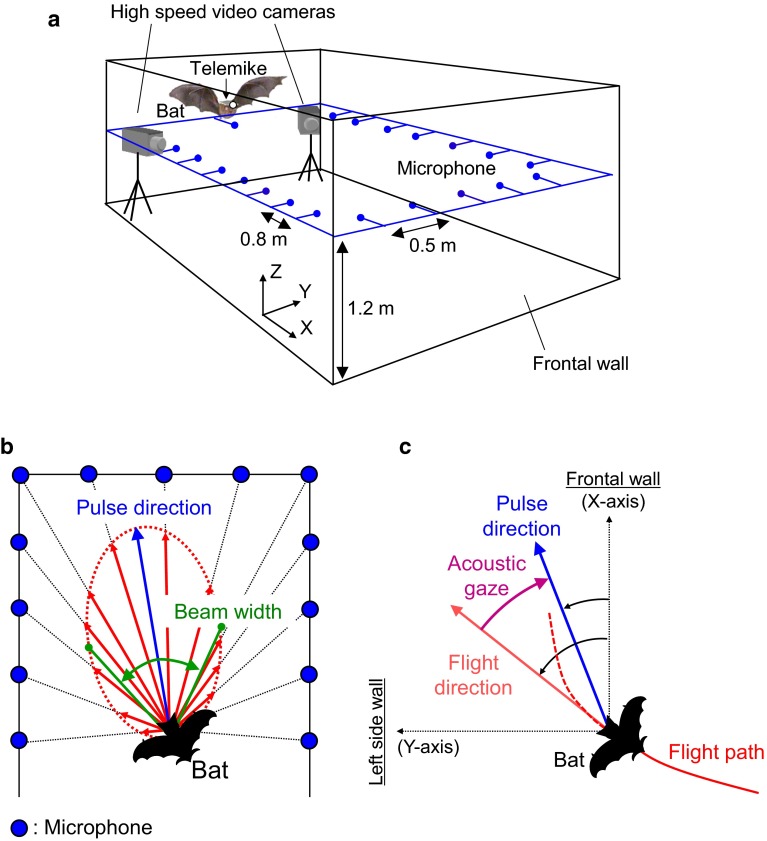
System for measuring echolocation pulses and flight trajectory of bats during flight. **a** Arrangement of the microphone array in the flight chamber. **b** Procedure for deriving horizontal pulse direction and beam width from microphone array recordings. The *blue arrow* indicates pulse direction, and the *green double*-*headed arrow* indicates the beam width of the pulse. **c** Acoustic gaze, defined as the angle between pulse direction and flight direction

Figure 2a shows a schematic diagram of the measurement system used in this study. The experiments were conducted in a flight chamber [8 m (L) × 3 m (W) × 2 m (H)] under red-filtered light (>650 nm) to avoid visual disturbance that might affect bats’ behavior. The flight chamber was constructed of steel plates to minimize interference from external electromagnetic signals from commercial FM radio stations.

All experiments were conducted using naïve bats within a week of capture. The behavior of each bat was observed only during its first flight in the chamber to investigate the use of echolocation during exploration of an unfamiliar setting. Data were thus obtained for one flight from each of seven *P. abramus* and six *R. ferrumequinum nippon* individuals. The experimenter carefully carried each bat into the flight chamber, holding it in his/her hands to prevent it from conducting echolocation to explore the chamber before recording began.

### Video recordings

Flights were recorded using two digital high-speed video cameras (IDT Japan, Inc., MotionPro X3, Tokyo, Japan; 125 frames per second) located in the left and right rear corners of the flight chamber to map the three-dimensional (3D) position of bats during flight. Cameras were placed behind the start position so that they would not interfere with the bat’s flight path; 3D flight paths were reconstructed from video footage using motion analysis software (Ditect Corporation, DIPPMotionPro ver.2.2.1.0, Tokyo, Japan). Before flights were filmed, a 3D reference frame was placed at known coordinates in the center of the flight chamber and briefly recorded by the two video cameras. The analysis software used the cameras’ stereo view of the reference frame to calibrate the 3D flight path reconstruction system. Based on a direct linear transformation technique using the coordinates of the reference frame, the successive positions of the flying bats, as well as the locations of other objects, were reconstructed from the pair of 2D video images. Using 3D coordinate data, the flight trajectory of each bat was determined, and a polynomial equation was fitted to the data to create a smooth flight path. The instant 3D flight direction of the bat was obtained, in conjunction with the acoustic characteristics of the echolocation sounds, from the 3D coordinates at a 125 frame rate.

### Sound recordings by an on-board telemetry microphone

A custom-made telemetry microphone (Telemike) was mounted on each subject to record the timing and amplitude of sounds emitted during flight. This recording procedure was the same as that used previously (Hiryu et al. 2008b; Matsuta et al. 2013). The Telemike consisted of a 1/8-inch omnidirectional condenser microphone (Knowles, Model FG-3629, Itasca, Illinois, USA), a miniature custom-designed FM transmitter unit, a 1.5-V hearing aid battery (Sony, Type SR521SW, Tokyo, Japan), and a transmitting antenna. The total weight of the Telemike was approximately 0.6 g. The Telemike was attached to the back of the bat with a piece of double-sided adhesive tape. The microphone pointed forward and was positioned approximately 1 cm above the mouth on *P. abramus* and 1 cm above the noseleaf on *R. ferrumequinum nippon*. The microphone was centered between the bat’s pinnae. An FM antenna (RadioShack Corporation, Model15-1859, TX, USA) suspended from the ceiling of the flight chamber received radio signals transmitted by the Telemike. The signals were demodulated to recover the bat’s ultrasonic broadcasts using a custom-made FM receiver, then band-pass filtered to a range of 20–150 kHz (NF Corporation, Model 3625, Yokohama, Japan), digitized by a Digital Audio Tape recorder (SONY, Model SIR-1000 W, Tokyo, Japan, 16-bit, *f*_s_ = 384 kHz), and synchronized with video using a control signal. This enabled us to match flight coordinates with sound recordings and to store these data as files on the hard disk of a personal computer. The total frequency response of the Telemike system was flat, within 4 dB and between 20 and 100 kHz.

### Sound recordings from the microphone array

The recording procedure for the microphone array was the same as that used previously by (Matsuta et al. 2013). To measure the horizontal beam width and direction of the pulse emitted by bats during flight, a 20-ch microphone array was set up in the walls surrounding the chamber on a horizontal (*X*–*Y*) plane, 1.2 m above the floor (Fig. 2a). Microphones were placed 0.8 m apart along the *X*-axis and 0.5 m apart along the *Y*-axis. The electrical design of the microphone array circuit board was the same as that used in the study cited above. We used 1/8-inch omnidirectional (±3 dB, from 0° to 90°) condenser microphones (Knowles, Model FG-3629, Itasca, Illinois, USA) for the array. Urethane acoustic absorption material (20 × 20 cm) was attached to the rear of each microphone to reduce unexpected echoes from the walls and ceiling of the chamber. The data recording system for microphone array signals was independent of the telemetry microphone recording system. All signals recorded by the microphone array system were digitized using two high-speed data acquisition cards (National Instruments, Model NI PXI-6250, Tokyo, Japan; 16-bit, *f*s = 200 kHz). The digitized signals were stored as files on the hard disk of a personal computer using a custom program in LABVIEW (NI, Model NI LABVIEW 8.0, Tokyo, Japan) beginning with the control signal that triggered and synchronized video recording. Microphone array data were thus synchronized with flight coordinates as well as with the sound recordings made by the Telemike.

### Sound analysis

#### Telemike recordings

**Fig. 3:**
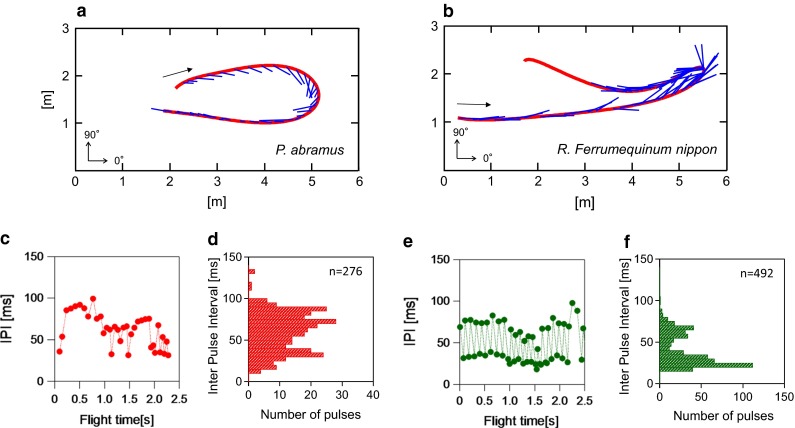
Echolocation of *P. abramus* and *R. ferrumequinum nippon* during first flights in the chamber. **a**, **b** *Top views* of flight trajectory (*red line*) and pulse directions (*blue lines*). **c**, **e** Changes in IPI as a function of flight time. **d**, **f** Histograms of IPI for all bats (seven *P. abramus* and six *R. ferrumequinum nippon*) during their first flight

Custom MATLAB routines were used to extract individual pulses from a spectrogram of Telemike recordings. The fundamental and second harmonic components of pulses were analyzed for *P. abramus* and *R. ferrumequinum nippon*, respectively, to determine the time at which bats emitted pulses. The interpulse interval (IPI) was defined as the interval between the onsets of successive calls. The energy maximum in the spectrogram of each component was measured to quantify changes in the sound pressure levels of pulses emitted during flight. These values are represented as solid lines with lengths proportional to the pulse pressure level that indicate the pulse direction along the flight path (e.g., blue lines in Fig. 3a).

#### Microphone array recording

The times at which emitted pulses arrived at each microphone of the array were estimated based on the bat’s 3D position and the time of pulse emission. This allowed us to use custom MATLAB routines to extract recorded pulses from the individual channels of the microphone array. The maximum energy of the fundamental or second harmonic component of each pulse was measured from the spectrograms extracted from individual channels in the microphone array. For *P. abramus*, the frequency at which the energy maximum appeared was defined as the peak frequency. In *R. ferrumequinum nippon*, the energy maximum appeared at the CF_2_ components of the pulses. However, the CF_2_ components were long in duration and overlapped with echoes from the surrounding walls in the microphone array recordings, making it inappropriate to quantify their sound pressure level (Matsuta et al. 2013). Therefore, a frequency 2 kHz below the CF_2_, which appeared within the terminal FM component of the second harmonic (tFM_2_), was designated as the peak frequency for *R. ferrumequinum nippon* and used to quantify changes in the sound pressure levels of pulses emitted by this species. The intra-individual variations in peak frequencies were 1–3 kHz for *P. abramus* and 1–2 kHz for *R. ferrumequinum nippon*. Such small variations in frequency are unlikely to affect the measurement of beam width.

The sound pressure levels of the pulses were then corrected for the propagation loss of sounds in the air between the bat and each microphone and the sensitivity differences among the microphones in the array. Sound absorption was calculated from measured absorption coefficients, which were determined for the average frequencies at the peak energy in the FM pulse of *P. abramus* (1.7 dB/m at 50 kHz) and the tFM_2_ component of *R. ferrumequinum nippon* (2.4 dB/m at 65 kHz). The sensitivity of the microphone array elements was measured by presenting tone bursts at 50 and 65 kHz to each microphone of the array (a 3-ms burst at 50 kHz for *P. abramus* and at 65 kHz for *R. ferrumequinum nippon*; 107 dB of sound pressure level (SPL) at 1 m from the loudspeaker) using an ultrasonic loudspeaker (PT-R7 III, Pioneer Corporation, Kanagawa, Japan), allowing recorded sounds to be calibrated according to sensitivity differences among the microphones.

For each emitted sound, the corrected sound pressure levels of each microphone within the array were converted into vectors, which were added to compute horizontal pulse directions (blue arrow, Fig. 2b) (Ghose and Moss 2006). Pulse direction was set as 0°, and the pattern of pulse directivity was fitted with a Gaussian shape using the corrected sound pressure vectors across all microphones for each pulse. Beam width was defined as the portion of the pulse directivity pattern between −6 dB (half-amplitude) off-axis angles from the pulse direction (green double-headed arrow, Fig. 2b).

The measurement errors in pulse direction and beam width created by our microphone array system were previously investigated using ultrasound tone bursts from a loudspeaker (PT-R7 III, Pioneer) set up in the chamber (Matsuta et al. 2013). Pulse direction and beam width were measured as the loudspeaker was moved between 0.5 and 6 m from the front wall. The measurement errors of the pulse direction and beam width were less than approximately 3° and 5° at a distance of 1 to 6 m from the chamber’s front wall. To prevent a vertical gradient in measurements of beam width, these measurements were taken between 2 and 6 m from the front wall. Across all flight sessions, the altitude difference between the bat and the microphone array (1.2 m above the floor) ranged from −0.6 to 0.2 m for seven *P. abramus* and from −0.6 to 0.5 m for six *R. ferrumequinum nippon*. This small interspecific difference in flight altitude is unlikely to affect the results of beam pattern comparisons.

The call parameters investigated in this study were the IPI, pulse direction, and beam width of the pulses emitted by each species of bat. IPI was measured from Telemike recordings, whereas analyses of pulse direction and beam width were conducted using recordings from the microphone array (as described above).

Figure 2c shows the definitions of the horizontal angular components in this study. We defined the directions of the *X*-axis and *Y*-axis as 0° and 90°, respectively, in the horizontal plane. The acoustic gaze was defined as the angular difference between pulse direction and flight direction (Ghose and Moss 2006). The sign (±) of the acoustic gaze is positive when the direction of the emitted pulse is counterclockwise from the flight direction, and it is negative when it is clockwise. In this study, we also measured the amount of absolute change in the acoustic gaze between successive pulses, ∆gaze, to quantify how much bats shifted their acoustic gaze between emissions when scanning the space. We investigated the interspecific difference between *P. abramus* and *R. ferrumequinum nippon* in acoustic gaze, ∆gaze and beam width. We used Student’s *t* test, *F*-test, the Mann–Whitney *U* test or the Levene test (as appropriate) to test for significant differences in call parameters between data sets.

## Results

### Echolocation of naïve bats

When an experimenter released an individual bat at one end of the flight chamber, the bat flew in a circular motion inside the chamber. The average maximum flight speeds of seven *P. abramus* and six *R. ferrumequinum nippon* during the first few circles of their first flights in the chamber were 2.9 ± 1.1 m/s (*n* = 7 flights) and 2.6 ± 0.8 m/s (*n* = 6 flights), respectively. Figure 3a shows a representative flight path (red line) and the pulse directions (blue solid lines) of the first flight of a naïve *P. abramus*. This individual emitted pulses along the inner periphery of the flight path while making a U-turn. As a result, the direction of pulses shifted smoothly according to flight direction. This flight, with pulses directed toward the inner periphery of the flight direction, was characteristic of all *P. abramus* subjects. In contrast, Fig. 3b shows that naïve *R. ferrumequinum nippon* subjects usually made dynamic changes in pulse direction, shifting pulse direction to the right and left of the flight direction, suggesting that *R. ferrumequinum nippon* individuals use a strategy for controlling acoustic gaze that differ from that used by *P. abramus* (see the next section).

Figure 3c and e show changes in IPIs during these first flights for *P. abramus* and *R. ferrumequinum nippon*, respectively. Double pulses, defined as two pulses with an IPI of less than 40 ms, are used differently by the two species. *R. ferrumequinum nippon* mostly emitted double pulses (i.e., based on all first-flight data), while 37 % (103/276 pulses) of *P. abramus* and 72 % (353/492 pulses) of *R. ferrumequinum nippon* broadcasts were emitted as double pulses. The mean percentages of double pulse usage of *R. ferrumequinum nippon* (mean ± SD = 73 ± 9 %, 6 bats) was significantly higher than that of *P. abramus* (42 ± 21 %, 7 bats) (Student’s *t*-test, *P* < 0.01). Figure 3d and f show histograms of IPIs for *P. abramus* and *R. ferrumequinum nippon* subjects during their first flights. In *P. abramus*, IPIs ranged from 10 to 100 ms (mean ± SD = 60 ± 23 ms, *n* = 276). In contrast, the histogram of *R. ferrumequinum nippon* IPIs shows two distinct peaks due to the prevalence of double pulses; the average IPI for *R. ferrumequinum nippon* was 41 ± 24 ms (*n* = 491). The peak around the shorter IPI (<40 ms), 26.3 ± 5.8 ms (*n* = 303), was significantly narrowly distributed, whereas the second peak, at the longer IPI (>40 ms), 62.3 ± 10.9 ms (*n* = 188), showed a broader distribution (ANOVA, *F*_187,302_ = 3.57, *P* < 0.001).

### Relationship between beam width and pulse direction

**Fig. 4:**
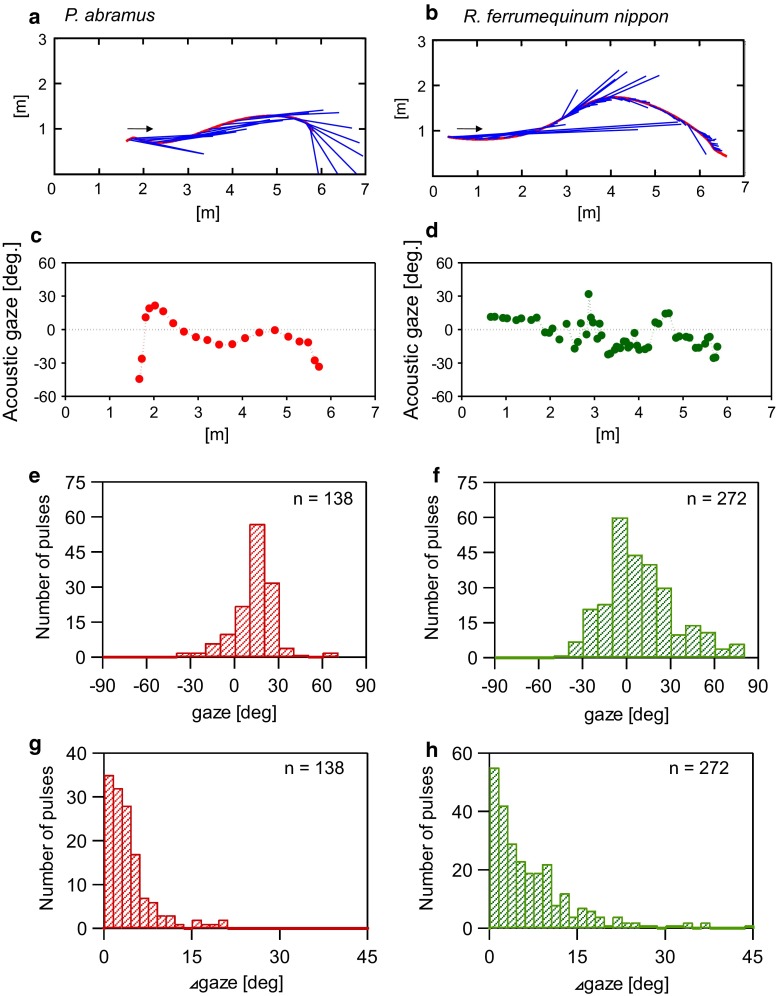
Comparison of acoustic gaze control between *P. abramus* and *R. ferrumequinum nippon* during the first flight. *Top views* of flight trajectories (*red line*) with pulse directions (*blue lines*) for representative *P. abramus* (**a**) and *R. ferrumequinum nippon* (**b**). **c**, **d** Angle of acoustic gaze during the flights shown in (**a**) and (**b**). **e**, **f** Distributions of acoustic gaze during flights of all bats. **g**, **h** Distributions of the amount of absolute change in acoustic gaze (∆gaze) between successive pulses in all bats

Figure 4 shows other examples of different individuals, illustrating differences in the acoustic gaze of naïve *P. abramus* and *R. ferrumequinum nippon* (note: the data represent only the one-way trip from the start to the frontal wall). As described above, *P. abramus* smoothly shifted acoustic gaze along the flight path (Fig. 4a, c), whereas *R. ferrumequinum nippon* frequently shifted acoustic gaze from side to side (see Fig. 4b, d). We compared the distributions of acoustic gazes of the two bat species (see Fig. 4e, f) and found that the average acoustic gaze fell between ~−20° and 30° (mean ± SD = 3.2 ± 14.6°) in *P. abramus* and between −40° and 60° (mean ± SD = −1.7 ± 24.2°) in *R. ferrumequinum nippon*. The acoustic gaze of *R. ferrumequinum nippon* was twice as wide as that of *P. abramus* and was composed of many more pulses (138 pulses from seven *P. abramus* vs 272 pulses from six *R. ferrumequinum nippon*; Levene test, *P* < 0.001). Furthermore, we calculated the absolute change in acoustic gaze (∆gaze) between successive pulses (Fig. 4g, h). The ∆gaze of *R. ferrumequinum nippon* was distributed up to approximately 15°–20° (*n* = 272, Fig. 4h) and differed significantly from that of *P. abramus* (Mann–Whitney *U* test, *P* < 0.001). In addition, although the ∆gaze within double pulses (IPI < 40 ms, *n* = 151) was not significantly different from that between double pulses (Mann–Whitney *U* test, *P* = 0.06), the ∆gaze was distributed up to approximately 15° (the maximum ∆gaze was 37°), suggesting that *R. ferrumequinum nippon* rapidly shifts its acoustic gaze even within double pulses.

**Fig. 5:**
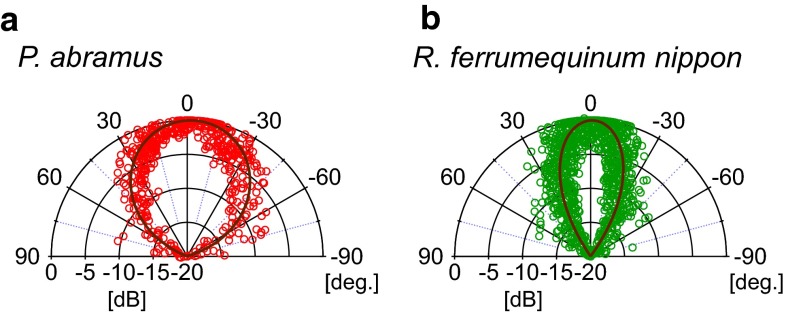
*Horizontal beam patterns* of echolocation pulses emitted by *P. abramus* (**a**) and *R. ferrumequinum nippon* (**b**) during first flights. Data were obtained from all bats (seven *P. abramus* and six *R. ferrumequinum*). *Solid line* shows the pattern of pulse directivity which was fitted with a Gaussian shape. The peak frequencies used when calculating *beam patterns* were 49.8 ± 3.0 kHz for *P. abramus* and 63.8 ± 2.2 kHz for *R. ferrumequinum nippon*

Peak frequencies across all flight sessions were 49.8 ± 3.0 kHz (*n* = 138 pulses) for *P. abramus* and 63.8 ± 2.2 kHz (*n* = 272 pulses) for *R. ferrumequinum nippon*. Figure 5a, b shows the beam patterns for each pulse at peak frequencies. The data depicted in Fig. 5 were taken only from the sounds emitted while bats approached the front wall during their first flight to avoid any measurement artifacts from the microphone array when constructing beam patterns. The resulting horizontal beam width (−6 dB off-axis angle from the pulse direction) was ± 38.3 ± 6.0° for *P. abramus* (*n* = 47 pulses) and ± 20.8 ± 6.0° for *R. ferrumequinum nippon* (*n* = 119 pulses). The beam width of *R. ferrumequinum nippon* was significantly narrower than that of *P. abramus* (Student’s *t* test, *P* < 0.001). We found that *R. ferrumequinum nippon* emitted narrower beams but shifted its acoustic gaze more widely and emitted pulses more frequently than *P. abramus* when flying in an unfamiliar space. In this way, *R. ferrumequinum nippon* compensates for its narrow beam width (half as wide as that of *P. abramus*) by shifting more often and over a wider angle than *P. abramus*.

## Discussion

### Compensation of the acoustic field of view

In this study, we hypothesized that the differences in the beam width of echolocation sounds between broadband FM and narrowband CF echolocating bat species affect the ways in which they control their acoustic gaze. When bats were tasked with scanning a novel spatial layout, they implemented species-specific behavioral strategies. *R. ferrumequinum nippon*, which emits long calls with narrower beams dominated by a single CF component, was found to shift its acoustic gaze more frequently than *P. abramus* which uses wider beams of brief FM sounds. We suggest that narrowband CF bats compensate for their narrower acoustic field of view, created by their high-frequency calls, by shifting their acoustic gaze further off-axis. Jakobsen et al. (2013) found that vespertilionid species compensate for their narrow directional beam due to different body sizes (emitter sizes) by changing the frequency of the echolocation sounds, which results in convergence of the acoustic field of view. From this perspective, compensation of the acoustic field of view is a common strategy among echolocating bats.

Based on general acoustic theory, the beam width of a bat’s broadband FM sounds will vary according to the frequency range (i.e., higher-frequency components have a narrower beam than those at lower frequencies) (Mogensen and Møhl 1979; Hartley et al. 1989). *Pipistrellus abramus* and some other FM bat species often emphasize sound energy in the low frequency range around the terminal frequency (TF), creating a quasi-CF portion following the initial FM sweep (Surlykke and Moss 2000; Hiryu et al. 2008a). Although there is no empirical evidence to show that FM bats use information from only the peak frequencies of their calls to perceive their environment, the dominant frequency of the returning echo is assumed to greatly affect the forming of the beam pattern, which restricts the acoustic field of view for target detection. From these points, we followed the example of previous studies, as we used a beam width at the peak frequency of the echolocation pulse (Jakobsen et al. 2013, 2015), which allowed us to conduct a simple comparison of the dominant spatial acoustical fields created by broadband FM and narrowband CF echolocators.

### Beam width vs acoustic gaze control

Echolocating bats are known to adapt their flight performance and sonar characteristics in response to their feeding ecology (i.e., Simmons and Stein 1980; Neuweiler 1984; Schnitzler and Kalko 2001; Fenton 2010; Kounitsky et al. 2015). *P. abramus* bats forage in open spaces for tiny prey, a few millimeters in size, using a brief broadband FM pulse with a central frequency of 40 kHz (Fujioka et al. 2011). On the other hand, *R. ferrumequinum nippon* individuals hunt moths by emitting long narrowband pulses. Because high-frequency sounds are subject to considerable Doppler shifting as a result of the fluttering of moths, *R. ferrumequinum nippon* have adapted to produce high-frequency narrowband CF sounds at 70 kHz, which are specialized for detecting Doppler shifts and are important for the feeding ecology of this species. This species-specific difference in the acoustic characteristics of echolocation sounds causes *P. abramus* and *R. ferrumequinum nippon* to form acoustic beams of different widths. This difference, in turn, prompts behavioral differences in acoustic gaze control, with the acoustic gaze of *R. ferrumequinum nippon* distributed significantly more wide than that of *P. abramus* during exploratory flight in an unfamiliar place (see Results, Levene test, *P* < 0.001).

*P. abramus* emits its echolocation calls through its mouth, whereas *R. ferrumequinum nippon* does so through its nostrils. The acoustic field of view of echolocating bats is affected by the emitter mechanism. The beam pattern of pulses emitted by oral-emitting bats can be estimated using a simple circular piston model, where the piston radius is equivalent to the size of bat’s open mouth (Strother and Mogus 1970; Mogensen and Møhl 1979; Hartley and Suthers 1987, 1989; Jakobsen and Surlykke 2010). Recent studies indicate that mouth-emitting broadband FM bats actively manipulate their acoustic field of view according to the situation by adjusting the gape of their mouths (Jakobsen et al. 2015; Kounitsky et al. 2015). Nasal-emitting bats must employ a different behavior. The beam width of sounds emitted from two closely spaced point sources, such as nostrils, changes with the ratio of the sound’s wavelength to the distance between the two sound sources. The nostril separations of CF bats are nearly equal to one-half the wavelength of the CF_2_ frequency, which enables them to configure the most appropriate beam pattern at the CF_2_ component (i.e., a main beam in the forward direction without a side lobe) (Möhres 1953; Strother and Mogus 1970; Schnitzler and Grinnell 1977; Hartley and Suthers 1987; Hiryu et al. 2006). In our previous study, we found that *R. ferrumequinum nippon* actively expands its beam width to retain a moving target in its spatial acoustic field of view during the final stages of capture, and it does so without changing the frequency of the emitted pulse (Matsuta et al. 2013). Although nasal emitters are thought to use their noseleaf and lancet to manipulate beam pattern (Hartley and Suthers 1987; Zhuang and Müller 2006, 2007; Vanderelst et al. 2010; He et al. 2015), the mechanism of such beam expansion has not yet been determined in nasal-emitting narrowband CF bats. The beam expansion has also been reported in bottlenose dolphins (*Tursiops truncatus*) during a target-detection task in which targets were placed in front of dolphins at increasingly greater off-axis angles (Moore et al. 2008). These studies suggest that active control of beam width based on the requirements of the task being performed is an important behavioral adaptation in animals that use biosonar. In this study, *R. ferrumequinum nippon* and *P. abramus* were not clearly observed performing such beam expansion during their first flight in an unfamiliar space. Instead, we found that CF bats also employ two additional methods to compensate for their inherently narrow echolocation beam: frequently shifting their acoustic gaze off-axis and emitting multiple pulses.

### Double pulses

Egyptian fruit bats produce tongue clicks with a dominant frequency of 30–35 kHz. When finding and landing on spherical objects in the dark, these bats point their acoustic gaze off-axis, alternately shifting to the left and the right so that the maximum slope of the beam as a function of angle to a target can be locked onto the target, which maximizes the sensitivity of target localization (Yovel et al. 2010). Such side-to-side scanning behavior has also been observed in *P. abramus* searching for prey during natural foraging (Fujioka et al. 2014). Off-axis scanning reduces the energy of echoes reflected from the target, but scanning to the left and right of an object expands the acoustic field of view.

We confirmed that *R. ferrumequinum nippon* emitted double pulses twice as often (72 %) as *P. abramus* (37 %) despite the fact that all bats were flown in the same unfamiliar flight space. *P. abramus* emitted double pulses only when closely approaching the wall, whereas *R. ferrumequinum nippon* emitted double pulses throughout the chamber. *R. ferrumequinum nippon* increased its sensing rate using paired pulses with a short IPI, suggesting that it can widen its narrow acoustical field of view not only by shifting its acoustic gaze but also by emitting double pulses in slightly different directions, expanding the total beam sight of the pulse pair. These bats thus employ both spatial and temporal mechanisms to control the flow of information from returning echoes when exploring a complex scene.

### Broadband FM and narrowband CF bats

Broadband FM and narrowband CF bats differ in a number of respects, such as the frequency structure of the echolocation sounds they emit and the environments in which they feed (Fenton et al. 2012). Narrowband CF bat species are also larger, with wider wing spans, which may cause a difference in their flight behavior. In this study, we restricted the flights of both types of bats to the same small room, and all were performed under unnatural echoic conditions. Therefore, we may suppose that the observed species-specific behavioral differences in gaze control will be even more pronounced under natural conditions. In future research, we plan to conduct a comparative study of the scanning behavior of narrowband CF (HDC) and broadband FM (LDC) bats during natural foraging.

## Abbreviations

CF: Constant frequency
CF_2_: Constant frequency component of the second harmonic
DSC: Doppler shift compensation
FM: Frequency modulated
iFM_2_: Initial frequency modulated component of the second harmonic
IPI: Interpulse interval
HDC: High duty cycle
LDC: Low duty cycle
TF: Terminal frequency
tFM_2_: Terminal frequency modulated component of the second harmonic

## References

[CR1] Fenton MB (2010). Convergences in the diversification of bats. Curr Zool.

[CR2] Fenton MB (2013). Questions, ideas and tools: lessons from bat echolocation. Anim Behav.

[CR3] Fenton MB, Faure PA, Ratcliffe JM (2012). Evolution of high duty cycle echolocation in bats. J Exp Biol.

[CR4] Fujioka E, Mantani S, Hiryu S, Riquimaroux H, Watanabe Y (2011). Echolocation and flight strategy of Japanese house bats during natural foraging, revealed by a microphone array system. J Acoust Soc Am.

[CR5] Fujioka E, Aihara I, Watanabe S, Hiryu S, Simmons JA, Riquimaroux H, Watanabe Y (2014). Rapid shifts of sonar attention by *Pipistrellus abramus* during natural hunting for multiple prey. J Acoust Soc Am.

[CR6] Ghose K, Moss CF (2003). The sonar beam pattern of a flying bat as it tracks tethered insects. J Acoust Soc Am.

[CR7] Ghose K, Moss CF (2006). Steering by hearing: a bat’s acoustic gaze is linked to its flight motor output by a delayed, adaptive linear law. J Neurosci.

[CR8] Ghose K, Horiuchi TK, Krishnaprasad PS, Moss CF (2006). Echolocating bats use a nearly time-optimal strategy to intercept prey. PLoS Biol.

[CR9] Grinnell AD, Schnitzler HU (1977). Directional sensitivity of echolocation in the horseshoe bat, *Rhinolophus ferrumequinum*. II: behavioral directionality of hearing. J Comp Physiol A.

[CR10] Hartley DJ, Suthers RA (1987). The sound emission pattern and the acoustical role of the noseleaf in the echolocating bat, *Carollia perspicillata*. J Acoust Soc Am.

[CR11] Hartley DJ, Suthers RA (1989). The sound emission pattern of the echolocatin bat, *Eptesicus fuscus*. J Acoust Soc Am.

[CR12] Hartley DJ, Suthers RA (1990). Sonar pulse radiation and filtering in the mustached bat, *Pteronotus parnellii rubiginosus*. J Acoust Soc Am.

[CR13] Hartley DJ, Campbell KA, Suthers RA (1989). The acoustic behavior of the fish-catching bat, *Noctilio leporinus*, during pre capture. J Acoust Soc Am.

[CR14] He W, Pedersen SC, Gupta AK, Simmons JA, Müller R (2015). Lancet dynamics in greater horseshoe bats, *Rhinolophus ferrumequinum*. PLoS One.

[CR15] Hiryu S, Katsura K, Lin LK, Riquimaroux H, Watanabe Y (2006). Radiation pattern of echolocation pulse in Taiwanese leaf-nosed bat, *Hipposideros terasensis*. Acoust Sci Technol.

[CR16] Hiryu S, Hagino T, Fujioka E, Riquimaroux H, Watanabe Y (2008). Adaptive echolocation sounds of insectivorous bats, *Pipistrellus abramus*, during foraging flights in the field. J Acoust Soc Am.

[CR17] Hiryu S, Shiori Y, Hosokawa T, Riquimaroux H, Watanabe Y (2008). On-board telemetry of emitted sounds from free-flying bats: compensation for velocity and distance stabilizes echo frequency and amplitude. J Comp Physiol A.

[CR18] Jakobsen L, Surlykke A (2010). Vespertilionid bats control the width of their biosonar sound beam dynamically during prey pursuit. Proc Natl Acad Sci USA.

[CR19] Jakobsen L, Ratcliffe JM, Surlykke A (2013). Convergent acoustic filed of view in echolocating bats. Nature.

[CR20] Jakobsen L, Olsen MN, Surlykke A (2015). Dynamics of the echolocation beam during prey pursuit in aerial hawking bats. Proc Natl Acad Sci USA.

[CR21] Kinoshita Y, Ogata D, Watanabe Y, Riquimaroux H, Ohta T, Hiryu S (2014). Prey pursuit strategy of Japanese horseshoe bats during an in-flight target-selection task. J Comp Physiol A.

[CR22] Kothari NB, Wohlgemuth MJ, Hulgard K, Surlykke A, Moss CF (2014). Timing matters: sonnar call groups facilitate target localization in bats. Front Physiol.

[CR23] Kounitsky P, Rydell J, Amichai E, Boonman A, Eitan O, Weiss AJ, Yovel Y (2015). Bats adjust their mouth gape to zoom their biosonar field of view. Proc Natl Acad Sci USA.

[CR24] Matsuta N, Hiryu S, Fujioka E, Yamada Y, Riquimaroux H, Watanabe Y (2013). Adaptive beam-width control of echolocation sounds by CF-FM bats, *Rhinolophus ferrumequinum nippon*, during prey-capture flight. J Exp Biol.

[CR25] Mogensen F, Møhl B (1979). Sound radiation patterns in the frequency domain of cries from a Vespertilionid bat. J Comp Physiol.

[CR26] Möhres FP (1953). Uber die ultraschallorientierung der hufeisennasen (Chiroptera-Rhinolophinae). Z Vergl Physiol.

[CR27] Moore PW, Dankiewicz LA, Houser D (2008). Beamwidth control and angular target detection in an echolocating bottlenose dolphin (*Tursiops truncatus*). J Acoust Soc Am.

[CR28] Moss CF, Surlykke A (2010). Probing the natural scene by echolocation in bats. Front Behav Neurosci.

[CR29] Moss CF, Bohn K, Gilkenson H, Surlykke A (2006). Active listening for spatial orientation in a complex auditory scene. PLoS Biol.

[CR30] Neuweiler G (1984). Foraging, echolocation and audition in bats. Naturwissenschaften.

[CR31] Schnitzler HU (1968). Die Ultraschallortungslaute der Hufeisen-Fledermäuse (Chiroptera-Rhinolophidae) in verschiedenen Orientierungssituationen [The ultrasonic sounds of horseshoe bats (Chiroptera-Rhinolophidae) in different orientation situations]. Z Vergl Physiol.

[CR32] Schnitzler HU, Denzinger A (2011). Auditory fovea and doppler shift compensation: adaptations for flutter detection in echolocating bats using CF-FM signals. J Comp Physiol A.

[CR33] Schnitzler HU, Grinnell AD (1977). Directional sensitivity of echolocation in the horseshoe bat, *Rhinolophus ferrumequinum*: I. Directionality of sound emission. J Comp Physiol A.

[CR34] Schnitzler HU, Kalko EKV (2001). Echolocation by insect-eating bats. Bioscience.

[CR35] Seibert A-M, Koblitz JC, Denzinger A, Schnitzler H-U (2013). Scanning behavior in echolocating common pipistrelle bats (*Pipistrellus pipistrellus*). PLoS One.

[CR36] Seibert A-M, Koblitz JC, Denzinger A, Schnitzler H-U (2015). Bidirectional echolocation in the bat *Barbastella barbastellus*: different signals of low source level are emitted upward through the nose and downward through the mouth. PLoS One.

[CR37] Shimozawa T, Suga N, Hendler P, Schuetze S (1974). Directional sensitivity of echolocation system in bats producing frequency-modulated signals. J Exp Biol.

[CR38] Simmons JA, Stein RA (1980). Acoustic imaging in bat sonar: echolocation signals and the evolution of echolocation. J Comp Physiol A.

[CR39] Strother GK, Mogus M (1970). Acoustical beam patterns for bats: some theoretical considerations. J Acoust Soc Am.

[CR40] Surlykke A, Moss CF (2000). Echolocation behavior of big brown bats, *Eptesicus fuscus*, in the field and the laboratory. J Acoust Soc Am.

[CR41] Surlykke A, Ghose K, Moss CF (2009). Acoustic scanning of natural scenes by echolocation in the big brown bat, *Eptesicus fuscus*. J Exp Biol.

[CR42] Surlykke A, Pedersen SB, Jakobsen L (2009). Echolocating bats emit a highly directional sonar sound beam in the field. Proc R Soc Lond B.

[CR43] Vanderelst D, De Mey F, Peremans H, Geipel I, Kalko E, Firzlaff U (2010). What noseleaves do for FM bats depends on their degree of sensorial specialization. PLoS One.

[CR44] Warnecke M, Lee W-J, Krishnan A, Moss CF (2016). Dynamic echo information guides flight in the big brown bat. Front Behav Neurosci.

[CR45] Wisniewska DM, Johnson M, Beedholm K, Wahlberg M, Madsen PT (2012). Acoustic gaze adjustments during active target selection in echolocating porpoises. J Exp Biol.

[CR46] Yovel Y, Falk B, Moss CF, Ulanovsky N (2010). Optimal localization by pointing off axis. Science.

[CR47] Zhuang Q, Müller R (2006). Noseleaf furrows in a horseshoe bat act as resonance cavities shaping the biosonar beam. Phys Rev Lett.

[CR48] Zhuang Q, Müller R (2007). Numerical study of the effect of the noseleaf on biosonar beamforming in a horseshoe bat. Phys Rev Lett.

